# Cytotoxic Effects of Bangladeshi Medicinal Plant Extracts

**DOI:** 10.1093/ecam/nep111

**Published:** 2011-01-12

**Authors:** Shaikh J. Uddin, I. Darren Grice, Evelin Tiralongo

**Affiliations:** ^1^School of Pharmacy, Griffith University, Gold Coast campus, 4222, Queensland, Australia; ^2^Institute for Glycomics, Griffith University, Gold Coast campus, Australia

## Abstract

To investigate the cytotoxic effect of some Bangladeshi medicinal plant extracts, 16 Bangladeshi medicinal plants were successively extracted with *n*-hexane, dichloromethane, methanol and water. The methanolic and aqueous extracts were screened for cytotoxic activity against healthy mouse fibroblasts (NIH3T3) and three human cancer-cell lines (gastric: AGS; colon: HT-29; and breast: MDA-MB-435S) using the MTT assay. Two methanolic extracts (*Hygrophila auriculata* and *Hibiscus tiliaceous*) and one aqueous extract (*Limnophila indica*) showed no toxicity against healthy mouse fibroblasts, but selective cytotoxicity against breast cancer cells (IC_50_ 1.1–1.6 mg mL^−1^). Seven methanolic extracts from *L. indica*, *Clerodendron inerme*, *Cynometra ramiflora*, *Xylocarpus moluccensis*, *Argemone mexicana*, *Ammannia baccifera* and *Acrostichum aureum* and four aqueous extracts from *Hygrophila auriculata*, *Bruguiera gymnorrhiza*, *X. moluccensis* and *Aegiceras corniculatum* showed low toxicity (IC_50_ > 2.5 mg mL^−1^) against mouse fibroblasts but selective cytotoxicity (IC_50_ 0.2–2.3 mg mL^−1^) against different cancer cell lines. The methanolic extract of *Blumea lacera* showed the highest cytotoxicity (IC_50_ 0.01–0.08 mg mL^−1^) against all tested cell lines among all extracts tested in this study. For some of the plants their traditional use as anticancer treatments correlates with the cytotoxic results, whereas for others so far unknown cytotoxic activities were identified.

## 1. Introduction

Natural products and related drugs are used to treat 87% of all categorized human diseases including bacterial infection, cancer and immunological disorders [1]. About 25% of prescribed drugs in the world originate from plants [2] and over 3000 species of plants have been reported to have anticancer properties [3]. About 80% of the population in developing countries rely on traditional plant based medicines for their primary health care needs [4]. Bangladesh has a rich and prestigious heritage of herbal medicines among the South Asian countries. More than 500 species of medicinal plants are estimated as growing in Bangladesh and about 250 species of them are used for the preparation of traditional medicines. However, the majority of these plants have not yet undergone chemical, pharmacological and toxicological studies to investigate their bioactive compound(s) [5]. Traditional records and ecological diversity indicate that Bangladeshi plants represent an exciting resource for possible lead structures in drug design.

In this study, 16 plants (*Adiantum caudatum, Ammannia baccifera, Argemone mexicana*, *Blumea lacera, Clerodendron inerme, Ficus religiosa, Hygrophila auriculata, Limnophila indica* and *Mollugo pentaphylla*) including seven mangrove species (*Acrostichum aureum, Aegiceras corniculatum, Bruguiera gymnorrhiza, Cynometra ramiflora, Hibiscus tiliaceous, Pandanus foetidus* and *Xylocarpus moluccensis*) were collected from tidal forests in the coastal Sundarbans (a swamp region in the Ganges delta) and other locations in the Khulna district of Bangladesh to screen them for possible cytotoxic activity. Except *Cynometra ramiflora*, all of these plants have been used in traditional medicine of Bangladesh for the treatment of various diseases such as cancer, inflammation or infectious diseases (Table 1) [5, 6]. Only limited research has been performed on these plants to evaluate their anticancer potential. In previous studies using extracts from *Hygrophila auriculata, Bruguiera gymnorrhiza, Clerodendron inerme, Blumea lacera*, *Hibiscus tiliaceous* and *Argemone mexicana* NFk-B inhibition, cytotoxic or cytoprotective activities have been observed [7–15]. For other plants (i.e., *Clerodendron inerme*, *M. pentaphylla* and *Aegiceras corniculatum*) anti-inflammatory activity [16–18], anti-oxidant activity (*Hygrophila auriculata, Bruguiera gymnorrhiza, X. moluccensis* and *Hibiscus tiliaceous*) [19–22] or antibacterial activity (*Adiantum caudatum, F. religiosa*, *M. pentaphylla* and *Argemone mexicana*) [17, 23–25] has been reported.

The majority of plant-based natural products are phenolic compounds [26]. Anticancer activity has been shown to be associated with a variety of classes, such as polyphenols, flavonoids and catechins [27]. A number of flavonoids and polyphenols have previously been isolated from different parts of *Hygrophila auriculata*, *L. indica*, *Bruguiera gymnorrhiza*, *Clerodendron inerme*, *Blumea lacera*, *Hibiscus tiliaceous, X. moluccensis* and *Aegiceras corniculatum* [5, 22, 28–36], which may be involved in their reported cytotoxic activity. Interestingly, no alkaloids, lectins or polysaccharides have been isolated to date from these plants, except an alkaloid from *Argemone mexicana* [37]. Here we report for the first time on the cytotoxic activity of methanolic and aqueous extracts from 16 Bangladeshi medicinal plants against normal mouse fibroblasts (NIH3T3), gastric cancer (AGS), colon cancer (HT29) and breast cancer (MDA-MB-435S) cells.

## 2. Methods

### 2.1. Plant Material

From March 2006 to May 2007, 16 plants were collected from tidal forests in the coastal Sundarbans (a swamp region in the Ganges delta), and other locations in the Khulna district of Bangladesh. The plant material was identified by the Bangladesh National Herbarium, Dhaka, Bangladesh and shade-dried. A specimen representing each collection was deposited in the Bangladesh National Herbarium, Dhaka, Bangladesh (Table 1).

### 2.2. Chemicals

*n*-Hexane, dichloromethane and methanol were purchased from Merck, Germany. Advanced Dulbecco's modified Eagle's medium (DMEM) (Batch #497466 and ID: Gibco 12491), newborn calf serum (NBCS) (Batch #1280182 and ID: Gibco 2901), trypsin-EDTA (Batch #475919 and ID: Gibco 25200) and l-glutamine (Batch #371023 and ID: Gibco 25030) were all obtained from Invitrogen, Australia. Dimethylsulfoxide (DMSO) (Batch #038K07101 and ID: Sigma D8418-100 mL), [3-(4,5-dimethylthiazol-2-yl)-2,5-diphenyltetrazolium bromide] (MTT) (Batch # 02317KH and ID: Sigma M2128-1G) were supplied from Sigma Aldrich, Germany.

### 2.3. Preparation of Extracts

The dried plant material (50–200 g) was ground into coarse powder and then successively extracted with solvents of decreasing lipophilicity (*n*-hexane, dichloromethane, methanol and milliQ-water) using a Soxhlet apparatus. The plant extracts were then filtered and the solvent was evaporated under reduced pressure followed by freeze-drying.

### 2.4. Cytotoxic Screening

#### 2.4.1. Cell Culture

Normal mouse fibroblast cells (NIH/3T3, ATCC: CRL-1658) and three human cancer cell lines gastric adenocarcinoma cells (AGS, ATCC: CRL-1739), colorectal adenocarcinoma cells (HT-29, ATCC: HTB-38) and breast ductal carcinoma cells (MDA-MB-435S, ATCC: HTB-129) were used for cytotoxicity screening of the Bangladeshi medicinal plant extracts. All cell lines were purchased from ATCC, Manassas, VA 20108, USA. Cell lines were cultured in Advanced DMEM supplemented with 10% inactivated NBCS and 5 mM l-glutamine, and grown at 37°C in a humidified atmosphere of 5% CO_2_ in air.

### 2.5. MTT Assay

The MTT [3-(4,5-dimethylthiazol-2-yl)-2,5-diphenyltetrazolium bromide] colorimetric assay developed by Mosmann [38], and further modified by Popiolkiewicz [39] and Kim [40], was used with minor modifications to screen for cytotoxic activity of Bangladeshi medicinal plant extracts. Briefly, the cells were seeded in 96-well plates at a density of 2.5 × 10^4^ to 3.5 × 10^4^ cells/well. Following 24-h incubation and attachment, the cells were treated with different concentrations of plant extract for 24 h. Following washing and incubation with MTT solution (0.5 mg mL^−1^ for cancer cell lines and 1 mg mL^−1^ for mouse fibroblasts) for 2 h, cells were lysed with DMSO. The absorbance was measured after 45 min using a microplate reader (Wallac 1420 Multilabel counter, PerkinElmer) at a wavelength of 560 nm. MilliQ-water and 0.75% DMSO served as the negative control for water and methanol extracts, respectively, while 25% DMSO served as the positive control. The MTT assay was validated using various concentrations of DMSO (0.25–25%).

Extracts showing cytotoxic activity were further tested at additional concentrations to calculate the IC_50_ values. The results are generated from two independent experiments; each experiment was performed in triplicate. The IC_50_ values were calculated with probit analysis software (LdP Line software, USA).

## 3. Results

A total of 32 extracts representing 16 Bangladeshi plant species from 16 plant families were screened for their cytotoxic activity against healthy mouse fibroblast and three human cancer cell lines (gastric, colon and breast cancer cells). The cytotoxic activities of the methanolic and aqueous extracts of the plants are summarized in Table 2. It is worth noting that IC_50_ values between 1 and 2 mg mL^−1^, while somewhat high, still point subtly towards selective activity. These “high" values are likely due to very low concentrations of compounds of interest, which would be considerably enriched upon bioactivity-guided fractionation.

### 3.1. Selective Cancer-Cell Cytotoxic Activity

Importantly, among the 32 extracts tested, three extracts, namely the methanolic extract of *Hygrophila auriculata* and *Hibiscus tiliaceous*, as well as the aqueous extract of *L. indica* showed no evident cytotoxicity against healthy mouse fibroblast cells, but selective cytotoxicity, particularly against breast cancer cells (IC_50_ 1.1–1.6 mg mL^−1^). Seven methanolic extracts (*Acrostichum aureum, Argemone mexicana, Ammannia baccifera, Clerodendron inerme, Cynometra ramiflora, L. indica, X. moluccensis*) and four aqueous extracts (*Aegiceras corniculatum, Bruguiera gymnorrhiza, Hygrophila auriculata, X. moluccensis*) showed low toxicity (IC_50_ > 2.5 mg mL^−1^) against mouse fibroblasts but selective cytotoxicity against different cancer cell lines. For example, the methanol extract from *Ammannia baccifera* leaves displayed selective cancer cell line cytotoxicity with IC_50_ values of 0.55, 0.59 and 0.91 mg mL^−1^ against gastric, colon and breast cancer cells, respectively. Similarly, the methanol extract from the pneumatophore of *X. moluccensis* showed IC_50_ values of 0.62 and 1.08 mg mL^−1^ against gastric and breast cancer cells, respectively. Moreover, the aqueous seed extract from *Hygrophila auriculata* displayed selective cancer cell cytotoxicity with an IC_50_ value of 0.22 mg mL^−1^ against colon cancer cells.

### 3.2. High Non-Selective Cytotoxic Activity

Four extracts showed cytotoxic activity against all tested cell lines including the healthy cell line. The methanolic extract of *Adiantum caudatum* leaves displayed moderate cytotoxcicity (IC_50_ 1.23–1.88 mg mL^−1^), whereas the aqueous extract from *Hibiscus tiliaceous* leaves showed significantly lower IC_50_ values, especially against gastric (IC_50_ 0.25 mg mL^−1^) and colon cancer cells (IC_50_ 0.8 mg mL^−1^). However, the methanolic extract from *Blumea lacera* leaves showed the highest cytotoxicity (IC_50_ 0.01–0.08 mg mL^−1^) against all tested cell lines among all extracts tested in this study.

### 3.3. Low or No Cytotoxic Activity

It should also be noted that 9 of the 16 aqueous Bangladeshi plant extracts show no or very low cytotoxic activity against healthy or cancer cell lines tested, whereas this is the case for only 3 of the 16 methanolic extracts. The low cytotoxic potential of the aqueous extracts is of great significance for their traditional use in the treatment of various disorders other than cancer.

## 4. Discussion

Complementary and alternative medicine (CAM) reports on multiple holistic approaches, including herbal medicines [41]. Recently, CAM has directed its interest towards therapies focused on important diseases throughout the world [42]. Drug discovery from natural sources is an area pertinent to CAM [43] and natural sources such as plants, animals and microorganisms provide a basis for the isolation of unique and potentially potent bioactive compounds [44]. Ethnopharmacologists can therefore provide CAM practitioners with relevant new information on therapies from natural sources [44]. This information helps to establish modern CAM treatment modalities, which may offer efficacious treatment to large populations affected with different diseases including cancer [45]. For example, a few studies into the anticancer potential of plants used in Bangladeshi folk medicine have been performed [46, 47].

Our study describes investigations into the anticancer potential of 16 so far not studied Bangladeshi medicinal plants by screening for cytotoxic activity against healthy mouse fibroblasts and three human cancer cell lines. Some plant extracts showed low or no toxicity against healthy mouse fibroblasts, but selective cytotoxicity against breast cancer cells, whereas others showed high cytotoxicity against all cell lines or were not cytotoxic against any of the cell lines tested.

Among the plant extracts that showed low toxicity against mouse fibroblasts but selective cytotoxicity against different cancer cell lines, the methanolic extract of *Acrostichum aureum* leaves showed the most potent selective cytotoxicity. Interestingly, in one study cytotoxic activity against HeLa cells has been reported for *Acrostichum aureum* [48].

The aqueous extracts from the seeds *Hygrophila auriculata*, with low toxicity against mouse fibroblasts and selective cytotoxicity against different cancer cell lines, has been previously been used as a traditional anticancer treatment [5]. Moreover, other *in vitro* studies have reported antioxidant (aerial parts), hepatoprotective (aqueous root extract), antitumor (petroleum ether root extract) and NFkB inhibition [9, 49] for extracts of *Hygrophila auriculata*.

In contrast, *Clerodendron inerme* is used for cancer prevention in the Indian traditional medical system [13]. Also, extracts of *Clerodendron inerme* have reported cytoprotective activity on oral squamous cells from 7,12-dimethylbenz[a]anthracene (DMBA) induced carcinogenesis [13]. Not surprisingly therefore, our study did not detect any significant cytotoxic effects of extracts from *Clerodendron inerme*.

The methanolic extracts from *Adiantum caudatum* and *Blumea lacera*, along with the aqueous extracts from *Blumea lacera* and *Hibiscus tiliaceous* showed high cytotoxicity against all cell lines tested, with the methanolic extract of *Blumea lacera* being the most cytotoxic amongst all tested plant extracts. Neither of these plants have previously been used as anticancer treatments in traditional Bangladeshi medicine, although, a hot aqueous extract of *Blumea lacera* has been reported to elicit cytotoxic activity against K562 cells (Human erythromyeloblastoid leukaemia cells) [7].

The methanolic extract of *Aegiceras corniculatum* showed very high cytotoxic activity against healthy, colon and breast cancer cells with IC_50_ values ranging from 0.02 to 0.66 mg mL^−1^, but very low cytotoxicity against gastric cancer cells. Interestingly, the plant *Aegiceras corniculatum* has been used traditionally as fish poison [32], however, no anticancer or cytotoxic activities have been reported to date.

Methanolic extracts of *Bruguiera gymnorrhiza* reported cytoprotective activity on bovine aortal endothelial cells (BAEC) against oxidized Low Density Lipoprotein (LDL) induced cytotoxicity [11]. Moreover, the petroleum ether extract (flowers) of *Bruguiera gymnorrhiza* has shown inhibitory activity for NFk-B and COX-2 [50]. In our study, aqueous and methanol extracts of *Bruguiera gymnorrhiza* leaves showed low to no cytotoxicity against any cell line, apart from the water extract, which displayed moderate selective cytotoxicity (IC_50_ 1.38 mg mL^−1^) against breast ductal carcinoma cells (MDA-MB-435S).

*Xylocarpus moluccensis* has been used traditionally in the treatment of swollen breasts [5]. More specific information is unfortunately unavailable; however, swollen breasts are usually a consequence of, hormonal changes, inflammation or benign or cancerous growth. Although it may not be directly related, it is interesting to note that in our study both extracts of *X. moluccensis* displayed moderate cytotoxic activity against breast ductal carcinoma cells.

This is the first time that aqueous and methanolic extracts from the 16 listed Bangladeshi plants (*Acrostichum aureum, Adiantum caudatum, Aegiceras corniculatum, Ammannia baccifera, Argemone mexicana*, *Blumea lacera, Bruguiera gymnorrhiza, Clerodendron inerme, Cynometra ramiflora, F. religiosa, Hibiscus tiliaceous, Hygrophila auriculata, L. indica*, *M. pentaphylla, P. foetidus*, and *X. moluccensis*) have been screened against human gastric, colon and breast cancer cell lines. This study supports the traditional uses of *Hygrophila auriculata*, *Clerodendron inerme*, and the reported cytotoxic activities of *Blumea lacera*, *Argemone mexicana* and *Acrostichum aureum*. Some of the plant extracts, such as *L. indica*, *Hibiscus tiliaceous*, *Cynometra ramiflora*, *Ammannia baccifera* and *Adiantum caudatum*, exerted selective cytotoxic activity, but neither cytotoxic activity had been reported previously, nor were the plants used traditionally in the treatment of cancer. This study provides an important basis for further investigation into the isolation, characterisation and mechanism of cytotoxic compounds from some of the screened Bangladeshi medicinal plants. Thus, these plants could be used as a source for new lead structures in drug design to combat cancer.

## Figures and Tables

**Table 1:** List of selected Bangladeshi medicinal plants with their traditional uses.

Plant species	Family	Local name	Voucher	Traditional uses
*Acrostichum aureum*	Ptridiaceae	Tiger fern	DACB 31538	R: rheumatism, treat wounds and boils; L: used to stop bleeding
*Adiantum caudatum*	Adiantaceae	Mayurshikha	DACB 31268	L: expectorant, antipyretic, diabetes, skin disease; WP: antibacterial, hypoglycaemic
*Aegiceras corniculatum*	Myrsinaceae	Kholisha	DACB 31584	B: fish poison, asthma, diabetes and rheumatism
*Ammannia baccifera*	Lythraceae	Jangli mendi	NA	L: rheumatism, skin diseases, ring worm and fever
*Argemone mexicana*	Papaveraceae	Shialkata	DACB 30213	L: antifungal, antiviral, antihelmintic, syphilitic infection, dysentery
*Blumea lacera*	Compositae	Kukursunga	NA	L: astringent, stimulant, antihelmintic, antimicrobial, anti-inflammatory and diuretic
*Bruguiera gymnorrhiza*	Rhizophoraceae	Kankra	DACB 31386	B: astringent, diarrhoea, stops bleeding; L: blood pressure
*Clerodendron inerme*	Verbenaceae	Bon Jui	DACB 31537	AP: hypotensive, fever; R: rheumatism, cancer prevention (India)
*Cynometra ramiflora*	Liguminosae	Kucha	NA	None reported
*Ficus religiosa*	Moraceae	Pan Bot	DACB 32004	B: antibacterial, astringent, diarrhoea, dysentery, gonorrhoea, antiprotozoal, antiviral and ulcers; L: skin disease
*Hibiscus tiliaceous*	Malvaceae	Bhola	DACB 31539	L: fever, coughs and dry throat; F: bronchitis, ear infections, dysentery, chest congestion
*Hygrophila auriculata*	Acanthaceae	Talmakna	DACB 31257	S: tonic, diarrhoea, dysentery, urinary discharge, gonorrhoea, diuretic, hepatoprotective; L: inflammation, rheumatism; AP: antineoplastic
*Limnophila indica*	Scrophulariaceae	Karpur	DACB 31536	AP: antiseptic, with coconut oil is used in elephantiasis, fever; WP: dysentery.
*Mollugo pentaphylla*	Molluginaceae	Khetpapra	NA^a^	L: antiseptic, used in digestion, relieve ear ache, spermicidal and antifungal
*Pandanus foetidus*	Pandanaceae	Kewa kata	DACB 31541	WP: leprosy, small pox, syphilis, scabies and heart and brain diseases; L: spadix and diabetes
*Xylocarpus moluccensis*	Meliaceae	Passur	DACB 31540	B: astringent, febrifuge, dysentery, diarrhoea; F: cure for elephantiasis and swelling of the breasts; S: itch

NA: not available; AP: aerial parts; B: bark; F: flowers; L: leaves; R: roots; S: seeds; WP: whole plant.

**Table 2:** Cytotoxic activity (IC_50_) of Bangladeshi plant extracts.

Species name	Part used	Extract	Yields (%)	Cytotoxic activity (IC_50_) ^a^(mg mL^−1^)			
				NIH/3T3	AGS	HT29	MDA-MB-435S
*Acrostichum aureum*	L	M	0.64	>2.50	1.02	>2.50	>2.50
		W	2.94	>2.50	>2.50	>2.50	>2.50
*Adiantum caudatum*	L	M	11.14	1.88	1.75	1.48	1.23
		W	8.04	>2.50	>2.50	>2.50	>2.50
*Aegiceras corniculatum*	B	M	6.39	0.02	>2.50	0.33	0.66
		W	1.40	>2.50	1.68	NC	1.91
*Argemone mexicana*	L	M	5.28	>2.50	>2.50	>2.50	1.82
		W	10.90	>2.50	>2.50	>2.50	>2.50
*Ammannia baccifera*	L	M	20.16	>2.50	0.55	0.59	0.91
		W	4.25	>2.50	>2.50	>2.50	>2.50
*Blumea lacera*	L	M	15.41	0.01	0.03	0.07	0.08
		W	16.15	0.67	0.99	0.48	0.39
*Bruguiera gymnorrhiza*	L	M	4.42	NC	>2.50	>2.50	>2.50
		W	2.15	>2.50	>2.50	>2.50	1.38
*Clerodendron inerme*	L	M	1.43	>2.50	2.38	>2.50	>2.50
		W	3.24	>2.50	>2.50	>2.50	>2.50
*Cynometra ramiflora*	B	M	6.16	>2.50	>2.50	1.79	2.35
		W	2.91	>2.50	>2.50	>2.50	>2.50
*Ficus religiosa*	L	M	1.14	1.01	2.16	>2.50	>2.50
		W	1.33	>2.50	NC	>2.50	>2.50
*Hibiscus tiliaceous*	L	M	4.84	NC	2.50	>2.50	1.14
		W	5.94	1.11	0.25	0.80	1.09
*Hygrophila auriculata*	S	M	0.51	NC	>2.50	>2.50	1.58
		W	4.36	>2.50	>2.50	0.22	1.40
*Limnophila indica*	L	M	11.14	>2.50	>2.50	2.19	1.24
		W	5.04	NC	2.24	NC	1.25
*Mollugo pentaphylla*	WP	M	6.22	>2.50	>2.50	>2.50	>2.50
		W	3.97	>2.50	>2.50	NC	>2.50
*Pandanus foetidus*	L	M	5.70	>2.50	NC	>2.50	>2.50
		W	4.45	NC	>2.50	>2.50	>2.50
*Xylocarpus moluccensis*	P	M	20.07	>2.50	0.62	>2.50	1.08
		W	5.42	>2.50	>2.50	>2.50	1.78

B: bark; L: leaves; S: seeds; WP: whole plant; P: pneumatophore; M: methanolic extract; W: aqueous extract; ^a^NC: no cytotoxicity at a concentration up to 2.5 mg mL^−1^; IC_50_ (50% inhibition of cell growth) calculated by probit analysis software, data was generated from two independent experiments, each experiment performed in triplicates.
